# Comparison of ARIMA, ETS, NNAR, TBATS and hybrid models to forecast the second wave of COVID-19 hospitalizations in Italy

**DOI:** 10.1007/s10198-021-01347-4

**Published:** 2021-08-04

**Authors:** Gaetano Perone

**Affiliations:** Department of Management, Economics and Quantitative Methods, University of Bergamo, via dei Caniana 2, 24127 Bergamo, Italy

**Keywords:** COVID-19, Outbreak, Italy, Hybrid forecasting models, ARIMA, NNAR, TBATS, C22, C53, I18

## Abstract

The coronavirus disease (COVID-19) is a severe, ongoing, novel pandemic that emerged in Wuhan, China, in December 2019. As of January 21, 2021, the virus had infected approximately 100 million people, causing over 2 million deaths. This article analyzed several time series forecasting methods to predict the spread of COVID-19 during the pandemic’s second wave in Italy (the period after October 13, 2020). The autoregressive moving average (ARIMA) model, innovations state space models for exponential smoothing (ETS), the neural network autoregression (NNAR) model, the trigonometric exponential smoothing state space model with Box–Cox transformation, ARMA errors, and trend and seasonal components (TBATS), and all of their feasible hybrid combinations were employed to forecast the number of patients hospitalized with mild symptoms and the number of patients hospitalized in the intensive care units (ICU). The data for the period February 21, 2020–October 13, 2020 were extracted from the website of the Italian Ministry of Health (www.salute.gov.it). The results showed that (i) hybrid models were better at capturing the linear, nonlinear, and seasonal pandemic patterns, significantly outperforming the respective single models for both time series, and (ii) the numbers of COVID-19-related hospitalizations of patients with mild symptoms and in the ICU were projected to increase rapidly from October 2020 to mid-November 2020. According to the estimations, the necessary ordinary and intensive care beds were expected to double in 10 days and to triple in approximately 20 days. These predictions were consistent with the observed trend, demonstrating that hybrid models may facilitate public health authorities’ decision-making, especially in the short-term.

## Introduction

The coronavirus disease (COVID-19) is a severe, ongoing, novel pandemic that officially emerged in Wuhan, China, in December 2019. As of January 21, 2021, it had affected 219 countries and territories with almost 100 million cases and over 2 million deaths [77]. At the time of writing, the countries most significantly affected include both advanced and developing countries, such as Brazil, France, India, Italy, Russia, Spain, the UK, and the US. From October to December 2020, several European countries, including Italy, saw a worrisome surge of COVID-19 infections.

Italy was the first European country to be severely impacted by COVID-19, and it remained one of the main epicenters of the pandemic for approximately 2 months, i.e., from mid-February 2020 to mid-April 2020. After that first peak, the pandemic curve progressively decreased until mid-August 2020. However, the spread of infection accelerated again in the late Summer and early Fall of 2020, and this second surge continues today. As of January 21, 2021, Italy has suffered 84,202 deaths and 2,428,221 cases.

The likelihood of new and consecutive COVID-19 waves is real, and efforts to study the pandemic’s trajectory are imperative to purchase medical devices and healthcare facilities and to manage health centers, clinics, hospitals, and ordinary and intensive care beds.

Thus, the first goal of this paper is to provide short-term and mid-term forecasts for the number of patients hospitalized with COVID-19 during the second wave of COVID-19 infections, i.e., during the period after October 13, 2020. COVID-19-related hospitalization trends offer a clear picture of the overall pressure on the national healthcare system. Moreover, models fitted to hospitalized patients are usually more reliable and accurate than models fitted to confirmed cases [30].^1^ The paper’s second goal is to compare and investigate the accuracy of several statistical methods.

In particular, I estimated four time series forecast techniques and all of their feasible hybrid combinations: the autoregressive moving average (ARIMA) model, innovations state space models for exponential smoothing (ETS), the neural network autoregression (NNAR) model, and the trigonometric exponential smoothing state space model with Box–Cox transformation, ARMA errors, and trend and seasonal components (TBATS).

The rest of this paper is organized as follows. “Related literature” reviews the relevant literature while “Materials and methods” presents the data used in the analysis and discusses the empirical strategy. “Evaluation metrics” presents the evaluation metrics used to measure the performance of the models. “Results and discussion” discusses the main findings and policy implications. Finally, “Conclusions” provides some conclusive considerations.

## Related literature

From the beginning of 2020, an increasing body of literature has employed various approaches to forecast the spread of the COVID-2019 outbreak [9, 22, 26, 58, 73, 78, 79, 83, 85]. The most frequently used were ARIMA models [3, 8, 14, 62], ETS models [13, 44], artificial neural network (ANN) models [55, 75], TBATS models [68, 71], models derived from the susceptible–infected–removed (SIR) basic approach [22, 26, 58, 78, 85], and hybrid models [15, 29, 68, 69]. The implementation and comparison of these approaches—with the exception of mechanistic–statistical models (such as SIR)—represents the core of this paper.

Ala’raj et al. [2] utilized a dynamic hybrid model based on a modified susceptible–exposed–infected–recovered–dead (SEIRD) model with ARIMA corrections of the residuals. They provided long-term forecasts for infected, recovered, and deceased people using a US COVID-19 dataset, and their model had a remarkable ability to make accurate predictions. Using a nonseasonal ARIMA model, Ceylan [14] made short-term predictions of cumulative confirmed cases after April 15, 2020, for France, Italy, and Spain. The forecasts showed low mean absolute percentage errors (MAPE) and seemed to be sufficiently reliable and suitable for the short-term epidemiological analysis of COVID-19 trends.

Hasan [29] proposed a hybrid model that incorporates ensemble empirical mode decomposition (EEMD) and neural networks to forecast real-time global COVID-19 cases for the period after May 18, 2020. The analysis showed that the ANN-EEMD approach was quite promising and outperformed traditional statistical methods, such as regression analysis and moving average.

Ribeiro et al. [65] provided short-term estimates of COVID-19 cumulative confirmed cases in Brazil by employing multiple approaches and selecting several models, such as ARIMA, cubist regression (CUBIST), random forest (RF), ridge regression (RIDGE), support vector regression (SVR), and stacking-ensemble learning (SEL). The models’ reliabilities were evaluated based on the improvement index, mean absolute error (MAE), and symmetric MAPE criteria. The analysis demonstrated that SVR and SEL performed best, but all models exhibited good forecasting performances.

Using ARIMA, TBATS, their statistical hybrid, and a mechanistic mathematical model combining the best of the previous models, Sardar et al. [68] attempted to forecast daily COVID-19 confirmed cases across India and in five different states (Delhi, Gujarat, Maharashtra, Punjab, and Tamil Nadu) from May 17, 2020, until May 31, 2020. The ensemble model showed the best prediction skills and suggested that COVID-19 that daily COVID-19 cases would significantly increase in the considered forecast window and that lockdown measures would be more effective in states with the highest percentages of symptomatic infection.

Wieczorek et al. [75] implemented deep neural network architectures, which learned by using a Nesterov-accelerated adaptive moment (Nadam) training model, to forecast cumulative confirmed COVID-19 cases in several countries and regions. The predictions, which referred to different time windows, revealed that the models had an extremely high level of accuracy (approximately 87.7% for most regions but, in some cases, reaching almost 100%).

Talkhi et al. [71] attempted to forecast the number of COVID-19 confirmed infections and deaths in Iran between August 15, 2020, and September 14, 2020, using several single and hybrid models. The extreme learning machine (ELM) and hybrid ARIMA–NNAR models were the most suitable for forecasting confirmed cases, while the Holt–Winters (HW) approach outperformed the others in predicting death cases.

Finally, Table 1 reports 30 international studies that utilized single or hybrid ARIMA, ETS, neural network, and TBATS models to forecast the transmission patterns of COVID-19 across the world.

**Table 1:** 30 selected international studies that utilized single or hybrid ARIMA, ETS, neural network, and TBATS models.

Authors	Data	Method	Country/region
Abotaleb [1]	Confirmed, deceased, and recovered	ARIMA and EGM	China, Italy, and the US
Ala’raj et al. [2]	Confirmed, deceased, and recovered	SEIRD-ARIMA	US
Alzahrani et al. [3]	Confirmed	ARIMA	Saudi Arabia
Aslam [4]	Active, confirmed, deceased, and recovered	KF-ARIMA, HW, and SutteARIMA	Pakistan
Awan and Aslam [5]	Confirmed	ARIMA	France, Germany, Italy, and Spain
Cao et al. [13]	Confirmed	ARIMA, ARIMAX, ETS, and SEIQDR	China
Ceylan [14]	Confirmed	ARIMA	France, Italy, and Spain
Chakraborty and Ghosh [15]	Confirmed	ARIMA–WBF	Canada, France, India, and South Korea
Dhamodharavadhani et al. [18]	Deceased	RBFNN, GRNN, NARNN, and PNN	India
Fantazzini [23]	Confirmed	ARIMA, ARIMAX, ETS, HVAR, SIR, and VAR	158 countries
Hasan [29]	Confirmed	ANN-EEMD	World (aggregate)
Ilie et al. [41]	Confirmed	ARIMA	nine countries
Joseph et al. [44]	Confirmed	ARIMA, ETS, INGARCH, and hybrid	nine countries
Katoch and Sindhu [45]	Confirmed	ARIMA	India
Kırbaş et al. [48]	Confirmed	ARIMA, LSTM, and NARNN	Eight European countries
Melin et al. [55]	Confirmed	ME-ANN	Mexico
Moftakhar and Seif [56]	Confirmed	ARIMA	Iran
Papastefanopoulos et al. [61]	Confirmed	ARIMA, DeepAR, FB, HWAAS, and N-Beats	10 countries
Perone [63]	Confirmed and deceased	ARIMA	Italy, Russia, and the US
Ribeiro et al. [65]	Confirmed	ARIMA, CUBIST, RF, RIDGE, SVR, and SEL	Brazil
Sardar et al. [68]	Confirmed	ARIMA, TBATS, hybrid, and mechanistic model	India
Sahai et al. [67]	Confirmed	ARIMA	Brazil, India, Russia, Spain, and the US
Singh et al. [69]	Deceased	ARIMA–WBF	France, Italy, Spain, the UK, and the US
Wang et al. [74]	Confirmed and deceased	ARIMA and ETS	India, Russia, the UK, and the US
Wieczorek et al. [75]	Confirmed	ANN	Many countries/regions
Yonar et al. [80]	Confirmed	ARIMA and B/W LES	G8 countries
Ganiny and Nisar [25]	Confirmed	ARIMA	India
Katris [46]	Confirmed	ARIMA, ETS, FFANN, MARS, their combinations, and SIR	Greece
Lee et al. [51]	Confirmed	ARIMA	South Korea
Talkhi et al. [71]	Confirmed and deceased	ARIMA, BSTS, ELM, HW, MLP, NNAR, Prophet, TBATS, and hybrid	Iran

*ANN* artificial neural network, *ARIMA* autoregressive integrated moving average, *ARIMAX* ARIMA with exogenous variables, *BSTS* Bayesian structural time-series, *B/W LES* Brown/Holt linear exponential smoothing method, *CUBIST* cubist regression, *DeepAR* probabilistic forecasting with autoregressive recurrent networks, *EEMD* ensemble empirical model decomposition, *EGM* exponential growth model, *ELM* extreme learning machines, *ETS* innovations state space models for exponential smoothing, *FB* Facebook’s prophet, *FFANN* feed-forward artificial neural network, *GRNN* generalized regression neural network, *HVAR* hierarchical vector autoregression, *HW* Holt–Winters method, *HWAAS* Holt–Winters additive model, *INGARCH* integer-valued generalized autoregressive conditional heteroskedastic, *KF* Kalman filter, *LSTM* long-short term memory, *MARS* multivariate adaptive regression splines, *ME-ANN* multiple ensemble artificial neural network, *MLP* multilayer perceptron, *NARNN* nonlinear autoregressive neural network, *N-Beats* neural basis expansion analysis, *NNAR* neural network autoregression, *PNN* probabilistic neural network, *RBFNN* radial basis function neural network, *RF* random forest, *RIDGE* ridge regression, *SEIQDR* susceptible–infected but undetected–infected quarantined–suspected–discharged, *SEIRD* susceptible–exposed–infected–recovered–dead, *SEL* stacking-ensemble learning, *SIR* susceptible–infected–recovered, *SutteARIMA* α-Sutte Indicator and ARIMA, *SVR* support vector regression, *TBATS* trigonometric exponential smoothing state space model with Box–Cox transformation, *ARMA errors* trend and seasonal components, *VAR* vector autoregression, *WBF* Wavelet-based forecasting

## Materials and methods

**Fig. 1:**
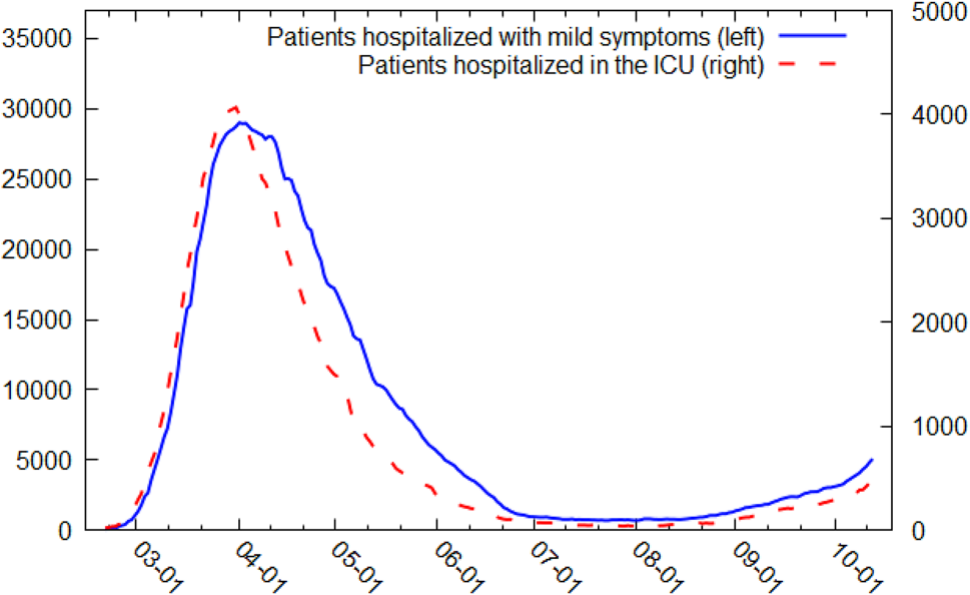
Patients hospitalized with mild symptoms and in the ICU from February 21, 2020 to October 13, 2020. Source: Italian Ministry of Health [43]

The data used in this article, which include 236 observations, referred to the real-time number of COVID-19 hospitalizations of patients with mild symptoms and patients assigned to the ICU in Italy from February 21, 2020, to October 13, 2020. I extracted the data from the official Italian Ministry of Health’s website (www.salute.gov.it). The confirmed COVID-19-related hospitalization trends appear in Fig. 1.

The data showed that the number of COVID-19 patients hospitalized with mild symptoms and the number of COVID-19 patients assigned to the ICU reached an initial peak on April 4, 2020. They then followed a downward trend until mid-August before accelerating again from the end of September 2020 to mid-October 2020. Recognizing that the use of only one model is never wise and may lead to unreliable forecasts [68], I computed the forecasts by employing different statistical techniques and their combinations. Specifically, linear ARIMA models, ETS models, linear and nonlinear NNAR models, TBATS models, and their feasible hybrid combinations were examined.

ARIMA models, which were first proposed by Box and Jenkins [11], represent one of the most widely used frameworks for epidemic/pandemic and disease time series predictions [66, 84]. Considering only the linear trend of a time series, they are able to capture both nonseasonal and seasonal patterns of that series. The following should be noted regarding the nonseasonal component of ARIMA models: (i) the autoregressive (AR) process aims to forecast a time series using a linear combination of its past values; (ii) the differencing (I) is required to make the time series stationary by removing (or mitigating) trend or seasonality (if any), and (iii) the moving average (MA) process aims to forecast future values using a linear combination of previous forecast errors. The seasonal component is similar to the nonseasonal component, but implies backshifts of the seasonal period, i.e., adding the seasonal parameters to the AR, I, and MA components, which allows the model to handle most of the seasonal patterns in the real-world data. Therefore, the final seasonal model can be denoted as ARIMA (*p*,*q*,*d*)(*P*,*Q*,*D*)_*m*_, where *m* is the seasonal period and the lowercase and uppercase letters indicate the number of nonseasonal and seasonal parameters for each of its three components, respectively [34], Sect. 8).

The ETS class of models was introduced in the late 1950s [12, 31, 76] to consider different combinations of trend and seasonal components. The basic ETS model consists of two main equations: a forecast equation and a smoothing equation. By integrating these two equations into an innovation state space model, which may correspond to the additive (A) or multiplicative (M) error assumption, it is possible to obtain an observation/measurement equation and a transition/state equation, respectively.^2^ The first equation describes the observed data while the second equation describes the behavior of the unobserved states. The states refer to the level, trend, and seasonality. The trend and seasonal components may be none (N), additive (A), additive damped (Ad),^3^ or multiplicative (M), resulting in a wide range of model combinations. The final model assumes the form of a three-character string (*Z*,*Z*,*Z*), where the first letter identifies the error assumption of the state space model, the second letter identifies the trend type, and the third letter identifies the season type. These models are able to produce a time series forecast by using the weighted average of its past values and adding more weight to recent observations [34], Sect. 7, [40].

NNAR models can be viewed as a network of neurons or nodes that depict complex nonlinear relationships and functional forms. In a basic neural network framework, the neurons are organized in two layers: (i) the bottom layer identifies the original time series, and (ii) the top layer identifies the predictions. The resulting model is equivalent to a simple linear regression and becomes nonlinear only when an intermediate layer with “hidden neurons” is included. For seasonal data, NNAR models can be described with the notation NNAR (*p*,*P*,*k*)_*m*_, where *m* is the seasonal period, *p* denotes the number of nonseasonal lagged inputs for the linear AR process, *P* represents the seasonal lags for the AR process, and *k* indicates the number of nodes/neurons in the hidden layer [34], Sect. 11.3).

Finally, TBATS models are a class of models that combine different approaches: trigonometric terms for modeling seasonality, Box–Cox transformation [10] for addressing heterogeneity, ARMA errors for addressing short-term dynamics, damping (if any) trends, and seasonal components. Therefore, TBATS models have several properties: (i) they deal well with very complex seasonal patterns, which might, for example, exhibit daily, weekly, and annual patterns simultaneously; (ii) they are able to consider time series nonlinear patterns, and iii) they can handle any type of autocorrelation in the residuals [34], Sect. 11.1, [71].

The combination of different times series forecast methods maximizes the chance of capturing seasonal, linear, and nonlinear patterns [60, 82] and is especially useful for predicting real-world phenomena, such as the COVID-19 pandemic, which are characterized by complex dynamics [7]. Well established from the seminal work of Bates and Granger [6], combining techniques with unique properties could allow models to achieve better performance and forecast accuracy.^4^ The models were calculated by using the following analytical procedures:

- ARIMA models were detected by applying the “auto.arima()” function included in the package “forecast” (in the R environment) and developed by Hyndman and Khandakar [35]. This function followed sequential steps to identify the best model, i.e., the number of *p* parameters of the autoregressive process (AR), the order *i* of differencing (I), the number of *q* parameters of the MA, and the number of the parameters of the seasonal component. It combined unit root tests^5^ and the minimization of the following estimation methods: the bias-corrected Akaike’s information criterion (AICc)^6^ and the maximum likelihood estimation (MLE). The unit root tests identified the order of differencing while the AICc and the MLE methods identified the order and the values of the parameters (respectively) of the seasonal and nonseasonal AR and MA processes;
- ETS models were identified by using the “ets()” function included in the package “forecast” (in the R environment) and developed by Hyndman et al. [39].^7^ In particular, I applied the Box–Cox [10] transformation to the data before estimating the model and then used the AICc metric to determine if the trend type was damped or not. The final three-character string identifying method (*Z*,*Z*,*Z*) was selected automatically;
- NNAR models were identified via the “nnetar()” function included in the package “forecast” (in the R environment) written by Hyndman [33].^8^ I proceeded as follows: (i) first, the Box–Cox transformation [10] was applied to the data before estimating the model; (ii) second, the optimal number of nonseasonal *p* lags for the AR(p) process was obtained by using the AICc metric; (iii) third, the seasonal *P* lags for the AR process were set to 1^9^; and (iv) finally, the optimal number of neurons was identified using the formula $k=\frac{(p+P+1)}{2}$ [34], Sect. 11.3);
- TBATS models were identified using the “tbats()” function included in the package “forecast” (in the R environment) as described in De Livera et al. [17]. The optimal Box–Cox transformation parameter, ARMA (*p*,*q*) order, damping parameter, and number of Fourier terms were selected using the Akaike’s information criterion (AIC) metric;^10^
- Hybrid models were identified via the “hybridModel()” function included in the “forecastHybrid” package (in the R environment) developed by Shaub and Ellis.^11^ The individual time series forecasting methods were combined as follows: (i) first, the Box–Cox power transformation [10] was applied to the inputs to increase the plausibility of the normality assumption; and (ii) then, the individual models were combined using both equal weights and cross-validated errors (“cv.errors”), which gave greater weight to the models that performed relatively better. In fact, since the best weighting procedure has not been established, I adopted a parsimonious approach and chose the one that performed better. Specifically, I tested the overall goodness-of-fit of all models with four common forecast accuracy measures: MAE, MAPE, mean absolute scaled error (MASE), and root mean square error (RMSE).

The estimated basic equation for the ARIMA was the following [16]:

1 $${\Delta }^{d}{y}_{t}={\phi }_{1}{\Delta }^{d}{y}_{t-1}+\dots {\phi }_{p}{\Delta }^{d}{y}_{t-p}+{\gamma }_{1}{\varepsilon }_{t-1}+\dots {\gamma }_{q}{\varepsilon }_{t-q}+{\varepsilon }_{t},$$

where ${\Delta }^{d}$ is the second difference operator, ${y}_{t}$ indicates the predicted values, *p* is the lag order of the AR process, $\phi$ is the coefficient of each parameters *p*, *q* is the order of the MA process, $\gamma$ is the coefficient of each parameter *q*, and ${\varepsilon }_{t}$ denotes the residuals of the errors at time *t*.

The estimated equations for the basic ETS (A,N,N) model with additive errors were the following [34], Sect. 7):

2 $$\mathrm{F}\mathrm{o}\mathrm{r}\mathrm{e}\mathrm{c}\mathrm{a}\mathrm{s}\mathrm{t}\;\mathrm{e}\mathrm{q}\mathrm{u}\mathrm{a}\mathrm{t}\mathrm{i}\mathrm{o}\mathrm{n}:{\widehat{y}}_{t+1|t}={l}_{t}.$$

3 $$\mathrm{S}\mathrm{m}\mathrm{o}\mathrm{o}\mathrm{t}\mathrm{h}\mathrm{i}\mathrm{n}\mathrm{g}\;\mathrm{e}\mathrm{q}\mathrm{u}\mathrm{a}\mathrm{t}\mathrm{i}\mathrm{o}\mathrm{n}:{l}_{t}={l}_{t-1}+\alpha \left({y}_{t}-{l}_{t-1}\right),$$

where ${l}_{t}$ is the new estimated level, ${\widehat{y}}_{t+1|t}$ denotes each one-step-ahead prediction for time *t*_+*1*_ which results from the weighted average of all the observed data, $0\le \alpha \le 1$ is the smoothing parameter, which controls the rate of decrease of the weights, and ${y}_{t}-{l}_{t-1}$ is the error at time *t*. Hence, each forecasted observation is the sum of the previous level and an error, and each type of error, additive or multiplicative, corresponds to a specific probability distribution. For a model with additive errors, as is this case here, errors are assumed to follow a normal distribution. Thus, Equations (2) and (3), respectively, can be rewritten as follows:

4 $$\mathrm{O}\mathrm{b}\mathrm{s}\mathrm{e}\mathrm{r}\mathrm{v}\mathrm{a}\mathrm{t}\mathrm{i}\mathrm{o}\mathrm{n}\;\mathrm{e}\mathrm{q}\mathrm{u}\mathrm{a}\mathrm{t}\mathrm{i}\mathrm{o}\mathrm{n}:{y}_{t}={l}_{t-1}+{\varepsilon}_{t},$$

5 $$\mathrm{T}\mathrm{r}\mathrm{a}\mathrm{n}\mathrm{s}\mathrm{i}\mathrm{t}\mathrm{i}\mathrm{o}\mathrm{n}\;\mathrm{e}\mathrm{q}\mathrm{u}\mathrm{a}\mathrm{t}\mathrm{i}\mathrm{o}\mathrm{n}:{l}_{t}={l}_{t-1}+\alpha {\varepsilon}_{t.}$$

Equations (4) and (5) represent the innovation state space models that underlie the exponential smoothing methods.

**Fig. 2:**
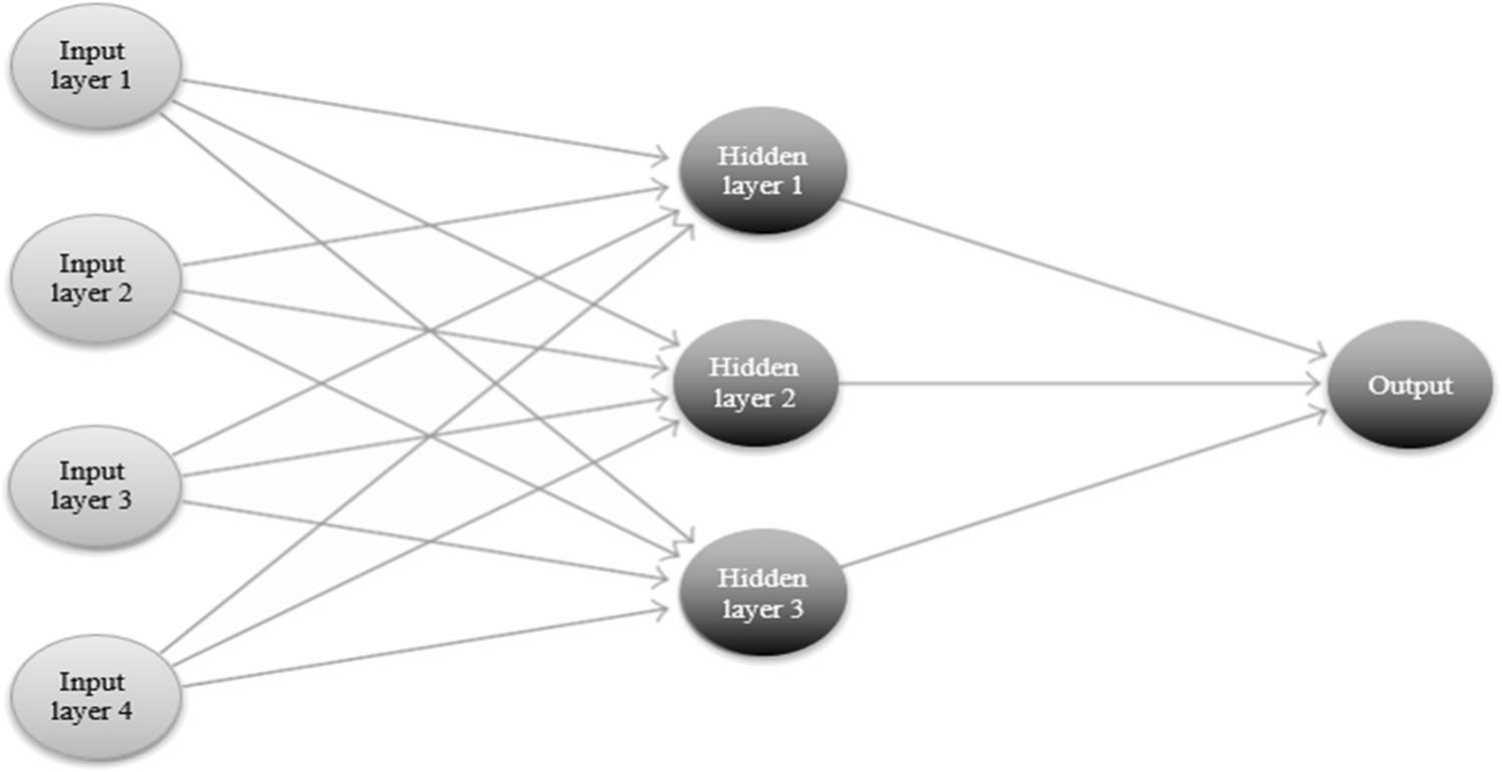
A neural network with four inputs and an intermediate layer with three hidden neurons

The basic form of the neural network autoregression equation was the following [34], Sect. 11.3):

6 $${y}_{t}=f\left({y}_{t-1}\right)+{\varepsilon}_{t},$$

where ${y}_{t}$ indicates the predicted values, ${y}_{t-1}{=\left({y}_{t-1,}{y}_{t-2}\dots ,{y}_{t-n}\right)}^{{'}}$ is a vector containing the lagged values of the observed data, *f* is the neural network with *n* hidden neurons in a single layer, and ${\varepsilon }_{t}$ is the error at time *t.* A simple graphical example of a nonlinear neural network is shown in Fig. 2.

Finally, the basic equation of the TBATS model took the following form [17]:

7 $${y}_{t}^{({\omega })}={\mathrm{l}}_{t-1}+\phi {b}_{t-1}+\sum _{i=1}^{T}{s}_{t-{m}_{i}}^{(i)}+{d}_{t},$$

where ${y}_{i}^{(\omega )}$ indicates the Box–Cox transformation parameter (*ω*) applied to the observation ${y}_{t}$ at time *t*, ${l}_{t}$ is the local level, $\phi$ is the damped trend, *b* is the long-run trend, *T* denotes the seasonal pattern, ${s}_{t}^{(i)}$ is the *i*th seasonal component,^12^${m}_{i}$ denotes the seasonal periods, and ${d}_{t}$ indicates an ARMA (p,q) process for residuals.

## Evaluation metrics

The main metrics used to compare the performances of the single and hybrid prediction models were MAE, MAPE, MASE, and RMSE. The formulae used to calculate each of these metrics appear below (Eqs. 8–11):

8 $$\mathrm{M}\mathrm{A}\mathrm{E}=\frac{1}{n}\sum _{i=1}^{n}\left|{y}_{i}-{\hat{y}}_{i}\right|,$$

9 $$\mathrm{M}\mathrm{A}\mathrm{P}\mathrm{E}=\frac{1}{n}\sum _{\;i=1}^{n}\frac{\left|{y}_{i}-{\hat{y}}_{i}\right|}{{y}_{i}}{*}100\mathrm{\%},$$

10 $$\mathrm{M}\mathrm{A}\mathrm{S}\mathrm{E}=\frac{1}{n}\sum _{i=1}^{n}\left(\frac{\left|{y}_{i}-{\hat{y}}_{i}\right|}{\frac{1}{n-1}{\sum }_{i=2}^{n}\left|{y}_{i}-{\hat{y}}_{i}-1\right|}\right),$$

11 $$\mathrm{R}\mathrm{M}\mathrm{S}\mathrm{E}=\sqrt{\frac{1}{n}\sum _{i=1}^{n}{\left({y}_{i}-{{\hat{y}}}_{i}\right)}^2},$$

where *n* represents the number of observations, ${y}_{i}$ denotes the actual values, and $\widehat{{y}_{i}}$ indicates the predicted values. Specifically, MAE and RMSE are both scale-dependent measures, although based on different errors. MAE is easier to interpret because minimizing it leads to predictions of the median, while minimizing RMSE leads to predictions of the mean. In fact, if the first metric is based on absolute errors, the second is based on squared errors. MAPE is probably the most widely employed error measure [27, 47], and unlike MAE and RMSE, it is not scale-dependent because it is based on percentage errors. Thus, it has the advantage of being a unit-free metric. However, it also requires some critical considerations. For example, it can lead to biased forecasts because it gives infinite or undefined results when one or more time series data point equals 0, and it puts a heavier penalty on negative errors (i.e., when predicted values are higher than actual values) than on positive errors. Finally, MASE, which was proposed by Hyndman and Koehler [36], is a scale-free error metric and probably the most versatile and reliable measure of forecast accuracy. It is superior to MAPE in that it does not give infinite or undefined values and can be used to compare forecast accuracy both on single and multiple time series.^13^ Since each model thus entails specific strengths and disadvantages, I opted for the prudent approach—evaluating the output of all of them.

## Results and discussion

Table 2 reports the best selected parameters for the single models^14^ while Tables 3 and 4 include the forecast accuracy measures for the single and hybrid models.^15^ For patients hospitalized with mild symptoms (Table 2), the optimal single models were the seasonal ARIMA (1,2,3) (0,0,1)_7_, ETS (A,Ad,N),^16^ NNAR (7,1,4)_7_, and TBATS (0.428, {2,2}, 1, {< 7,2 >}).^17^ For patients hospitalized in the ICU (Table 2), the optimal single models were the seasonal ARIMA (1,2,2)(0,0,1)_7_, ETS (A,A,N),^18^ NNAR (6,1,4)_7_, and (T)BATS (0.427,{0,0},1,−).^19^ The hybrid models were derived by combining the optimal single models with equal weights, which proved to be more suitable than weights based on error values.^20^

**Table 2:** Structure of the single models for patients hospitalized with mild symptoms and in the ICU

Models	AICc	Structure
Patients hospitalized with mild symptoms		
ARIMA	3170.61	Seasonal (1, 2, 3) (0, 0, 1)_7_
ETS	3846.92	(A, Ad, N)
NNAR	–	(7, 1, 4)_7_
TBATS	3550.91	(0.428, {2,2}, 1, {< 7,2 >})
Patients hospitalized in the ICU		
ARIMA	2106.46	Seasonal (1, 2, 2) (0, 0, 1)_7_
ETS	2775.88	(A, A, N)
NNAR	–	(6, 1, 4)_7_
(T)BATS	2506.27	(0.427, {0,0}, 1, –)

Notes: TBATS models were chosen using AIC metric

**Table 3:** Forecast accuracy measures for the single and hybrid models (patients hospitalized with mild symptoms)

Models	MAE	MAPE	MASE	RMSE	ACF1
ARIMA	116.2616	2.6125	0.0631	204.8225	− 0.0064
ETS	121.7029	4.3186	0.066	219.6733	0.0787
NNAR	111.0185	1.9759	0.0602	195.4126	− 0.051
TBATS	115.428	2.8267	0.0626	204.2566	0.1902
A–E	115.0882	3.4033	0.0624	207.1205	0.0234
A–N	105.4098	2.1634	0.0572	184.3577	− 0.0706
A–T	113.5426	2.6625	0.0616	200.8143	0.0803
E–N	107.3794	2.1219	0.0583	191.0452	− 0.037
E–T	114.7241	3.4622	0.0622	204.5928	0.0886
N–T	104.7705	2.0966	0.0568	183.9314	0.0228
A–E–N	108.655	2.1419	0.059	193.672	− 0.0355
A–E–T	113.0137	3.1372	0.0613	202.3212	0.0553
A–N–T	106.6527	2.1255	0.0579	189.0073	0.0579
E–N–T	108.3242	2.0805	0.0588	192.7942	0.0146
A–E–N–T	108.8197	2.1105	0.059	194.1662	0.006

Notes: *A* ARIMA, *E* ETS, *N* NNAR, *T* TBATS. Hybrid models were combined using equal weights

**Table 4:** Forecast accuracy measures for the single and hybrid models (patients hospitalized in the ICU)

Models	MAE	MAPE	MASE	RMSE	ACF1
ARIMA	12.5828	3.5411	0.0495	21.1697	0.0375
ETS	13.3832	3.59	0.0527	22.8157	0.0181
NNAR	11.6316	2.8082	0.0458	19.2895	− 0.1636
TBATS	14.226	3.5832	0.056	24.2076	0.3122
A–E	12.5388	3.47	0.0493	21.5479	0.0072
A–N	11.4917	3.0443	0.0452	18.7476	− 0.0513
A–T	12.8579	3.4874	0.0506	21.953	0.159
E–N	11.9035	3.064	0.0468	19.5496	− 0.0947
E–T	13.3764	3.5436	0.0526	22.9583	0.1504
N–T	11.9856	2.9767	0.0472	19.695	0.0627
A–E–N	11.8861	3.0595	0.0468	19.8477	− 0.0521
A–E–T	12.4876	3.3605	0.0491	21.3983	0.1502
A–N–T	12.0046	3.009	0.0472	19.9841	0.0474
E–N–T	12.383	3.0362	0.0487	20.7628	0.0377
A–E–N–T	12.188	3.0256	0.048	20.538	0.0277

Notes: *A* ARIMA, *E* ETS, *N* NNAR, *T* TBATS. Hybrid models were combined using equal weights

In particular, for patients hospitalized with mild symptoms, the most accurate single model was NNAR, followed by seasonal ARIMA, while the best hybrid model was NNAR–TBATS, followed by ARIMA–NNAR, and ARIMA–NNAR–TBATS. For patients hospitalized in the ICU, the best single model was NNAR, followed by ARIMA, while the best hybrid model was ARIMA–NNAR, followed by ARIMA–ETS–NNAR, and ETS–NNAR.^21^

Autocorrelation function (ACF) indicated that current values were not correlated with previous values at lag 1 (Tables 2 and 3). In fact, the correlation coefficient between one point and the next in the time series ranged from − 0.07 to 0.19 for patients hospitalized with mild symptoms and from − 0.16 to 0.31 for patients in the ICU. The highest values (0.19 and 0.31) were obtained for the TBATS model.^22^

According to Lewis’ [52] interpretation, since MAPE was always significantly lower than 10, all predictive models can be considered as highly accurate. Moreover, MASE was much lower than 1 for all models; therefore, all the proposed forecasting approaches performed significantly better than the forecasts from the (no-change) “naïve” methods, i.e., the forecasts with no adjustments for casual factors (Hyndman and Koehler 2006), which justifies the use of more complex and sophisticated models.

Tables 5 and 6 compare the hybrid models with the respective single models considering the minimization of MAE, MAPE, MASE, and RMSE metrics. For patients hospitalized with mild symptoms, the hybrid models outperformed the respective single models in 98 out of 112 metrics, i.e., on 87.5% of all the forecast accuracy measures. For patients hospitalized in the ICU, the hybrid models outperformed the respective single models in 81 out of 112 metrics, i.e., on 72.3% of all measures. In the latter case, however, almost all losses of efficiency (25 out 31) were attributable to NNAR.^23^ Thus, the hybrid models generally increased forecast accuracy, but this increase was more evident for patients hospitalized with mild symptoms.

**Table 5:** Comparison between hybrid models and respective single models considering the minimization of MAE, MAPE, MASE, and RMSE metrics (in percentage), for patients hospitalized with mild symptoms

Hybrid	Single	MAE	MAPE	MASE	RMSE
A–E	ARIMA	− 1.01	30.26	− 1.11	1.12
	ETS	− 5.44	− 21.19	− 5.45	− 5.71
A–N	ARIMA	− 9.33	− 17.19	− 9.35	− 9.99
	NNAR	− 5.05	9.49	− 4.98	− 5.66
A–T	ARIMA	− 2.34	1.91	− 2.38	− 1.96
	TBATS	− 1.63	− 5.81	− 1.6	− 1.69
E–N	ETS	− 11.77	− 50.87	− 11.67	− 13.03
	NNAR	− 3.28	7.39	− 3.16	− 2.23
E–T	ETS	− 5.73	− 18.83	− 5.76	− 6.86
	TBATS	− 0.6	22.48	− 0.64	0.16
N–T	NNAR	− 5.63	6.11	− 5.65	− 5.88
	TBATS	− 9.23	− 25.83	− 9.27	− 9.95
A–E–N	ARIMA	− 6.54	− 18.01	− 6.5	− 5.44
	ETS	− 10.72	− 50.4	− 10.61	− 11.84
	NNAR	− 2.135	8.4	− 1.99	− 0.89
A–E–T	ARIMA	− 2.79	20.08	− 2.85	− 1.22
	ETS	− 7.14	− 27.36	− 7.12	− 7.9
	TBATS	− 2.09	10.98	− 2.08	− 0.95
A–N–T	ARIMA	− 8.26	− 18.64	− 8.24	− 7.72
	NNAR	− 3.93	7.57	− 3.82	− 3.28
	TBATS	− 7.6	− 24.81	− 7.51	− 7.47
E–N–T	ETS	− 10.99	− 51.82	− 10.91	− 12.24
	NNAR	− 2.43	5.29	− 2.33	− 1.34
	TBATS	− 6.15	− 26.4	− 6.07	− 5.61
A–E–N–T	ARIMA	− 6.4	− 19.22	− 6.5	− 5.2
	ETS	− 10.59	− 51.13	− 10.61	− 11.61
	NNAR	− 1.98	6.81	− 1.99	− 0.64
	TBATS	− 5.73	− 25.34	− 5.75	− 4.94

Notes: *A*  ARIMA, *E* ETS, *N* NNAR, *T* TBATS. Negative (positive) values show the percentage efficiency gain (loss) from using hybrid models

**Table 6:** Comparison between hybrid models and respective single models considering the minimization of MAE, MAPE, MASE, and RMSE metrics (in percentage), for patients hospitalized in the ICU

Hybrid	Single	MAE	MAPE	MASE	RMSE
A–E	ARIMA	− 0.35	− 2.01	− 0.4	1.79
	ETS	− 6.31	− 3.34	− 6.45	− 5.56
A–N	ARIMA	− 8.67	− 14.03	− 8.69	− 11.44
	NNAR	− 1.2	8.41	− 1.31	− 2.81
A–T	ARIMA	2.19	− 1.52	2.22	3.7
	TBATS	− 9.62	− 2.67	− 9.64	− 9.31
E–N	ETS	− 11.06	− 14.65	− 11.2	− 14.32
	NNAR	2.34	9.11	2.18	1.35
E–T	ETS	− 0.05	− 1.29	− 0.19	0.63
	TBATS	− 5.97	− 1.11	− 6.07	− 5.16
N–T	NNAR	3.04	6	3.06	2.1
	TBATS	− 15.75	− 16.93	− 15.71	− 18.64
A–E–N	ARIMA	− 5.54	− 13.6	− 5.45	− 6.24
	ETS	− 11.19	− 14.78	− 11.2	− 13.01
	NNAR	2.19	8.95	2.18	2.89
A–E–T	ARIMA	− 0.76	− 5.1	− 0.81	1.08
	ETS	− 6.69	− 6.39	− 6.83	− 6.21
	TBATS	− 12.22	− 6.22	− 12.32	− 11.61
A–N–T	ARIMA	− 4.6	− 15.03	− 4.65	− 5.6
	NNAR	3.21	7.15	3.06	3.6
	TBATS	− 15.62	− 16.02	− 15.71	− 17.45
E–N–T	ETS	− 7.47	− 15.43	− 7.59	− 9
	NNAR	6.46	8.12	6.33	7.64
	TBATS	− 12.96	− 15.27	− 13.04	− 14.23
A–E–N–T	ARIMA	− 3.14	− 14.56	− 3.03	− 2.98
	ETS	− 8.93	− 15.72	− 8.92	− 9.98
	NNAR	4.78	7.74	4.8	6.47
	TBATS	− 14.33	− 15.56	− 14.29	− 15.16

Notes: *A*  ARIMA, *E* ETS, *N* NNAR, *T* TBATS. Negative (positive) values show the percentage efficiency gain (loss) from using hybrid models

**Fig. 3:**
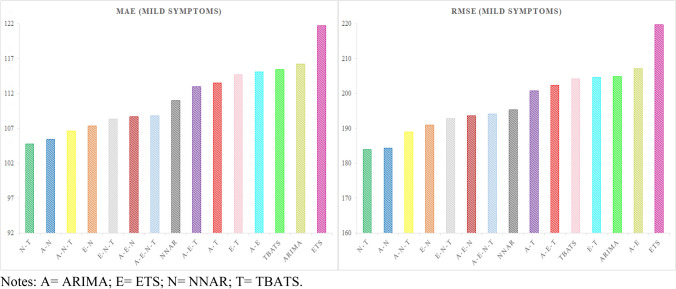
Models ranked by the MAE and RMSE metrics for patients hospitalized with mild symptoms

**Fig. 4:**
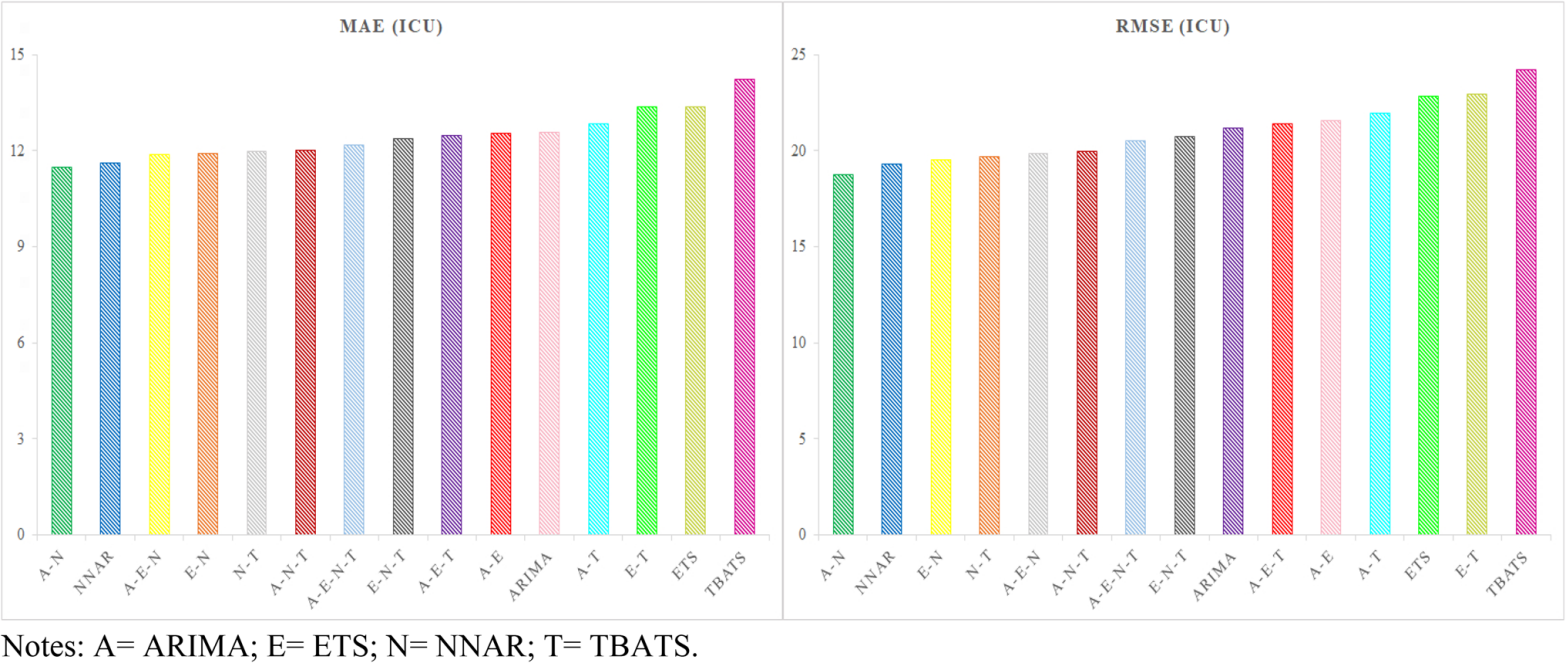
Models ranked by the the MAE and RMSE metrics for patients hospitalized in the ICU

The best hybrid model for patients hospitalized with mild symptoms—NNAR–TBATS—outperformed the single NNAR and TBATS models by 5.63–9.23% on MAE and 5.88–9.95% on RMSE. While, the best hybrid model for patients hospitalized in the ICU—ARIMA–NNAR—outperformed the single ARIMA and NNAR models by 1.2–8.67% on MAE, and 2.81–11.44% on RMSE. Figures 3 and 4 graphically represent the models for both time series ranked by the MAE and RMSE metrics.

**Fig. 5:**
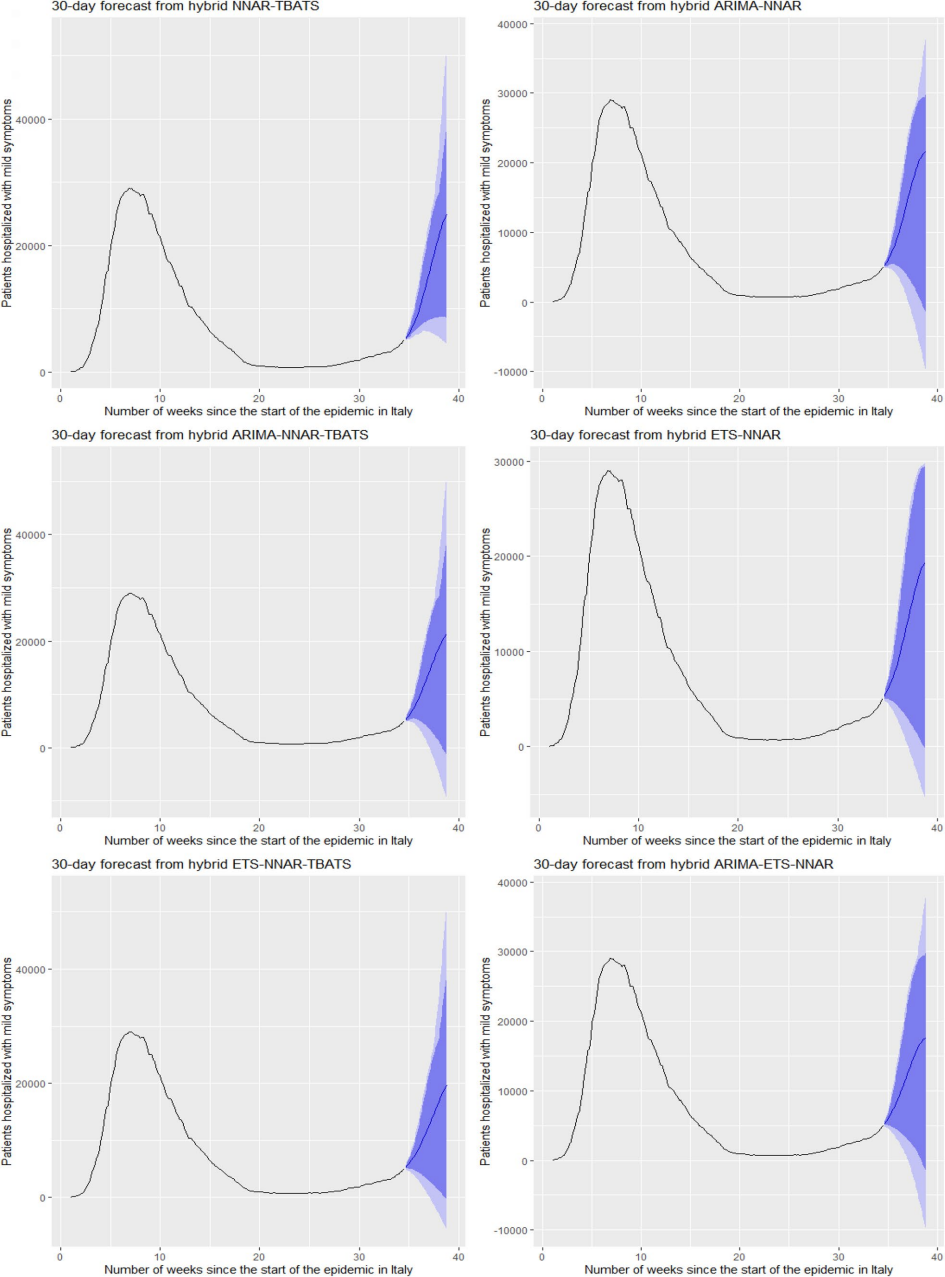
The six best forecast models for predicting patients with mild symptoms. Notes: the models were ranked (from first to sixth place) on the MAE metric

**Fig. 6:**
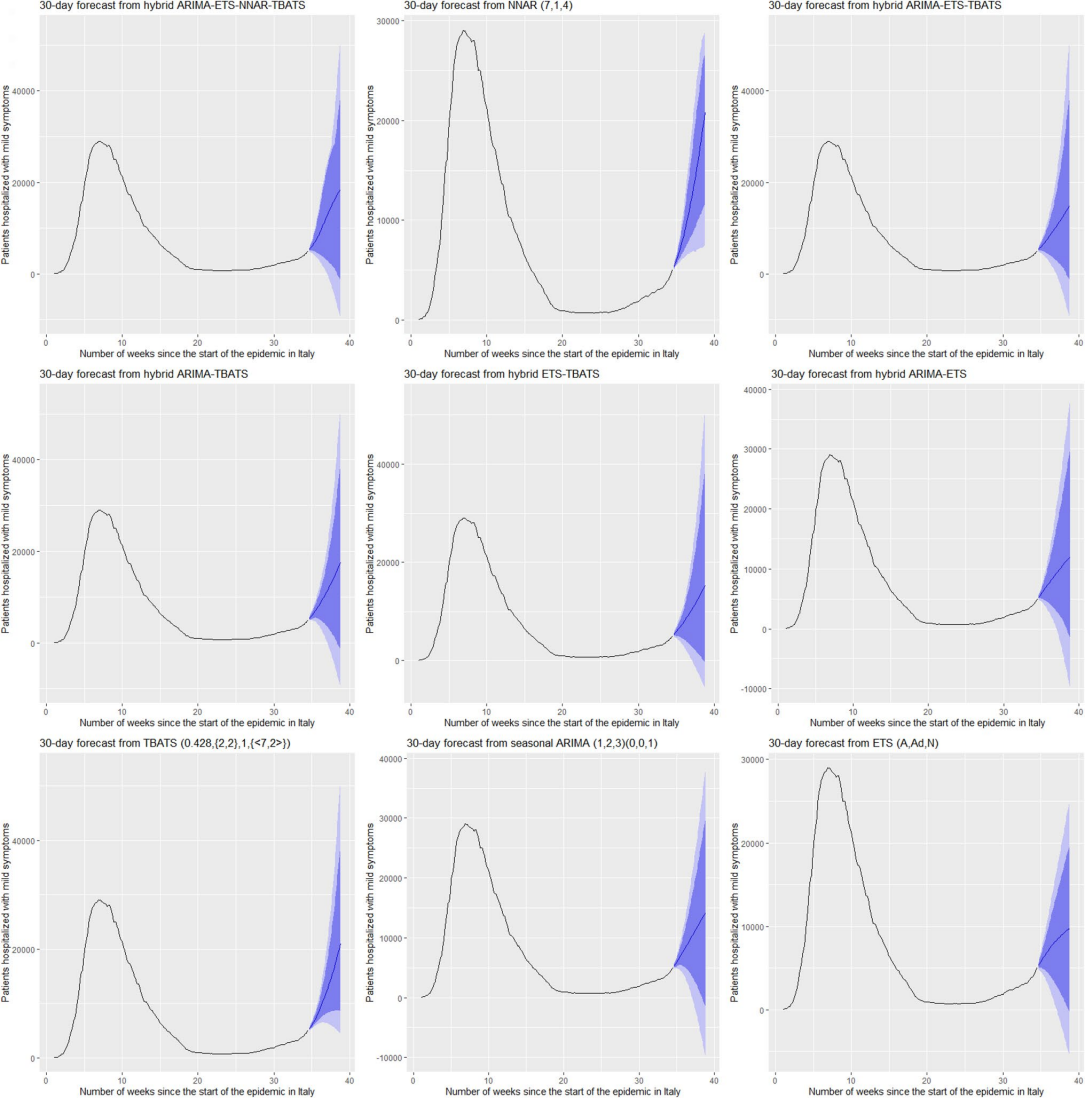
The remaining nine forecast models for predicting patients hospitalized with mild symptoms. Notes: The models were ranked (from seventh to fifteenth place) on the MAE metric

**Fig. 7:**
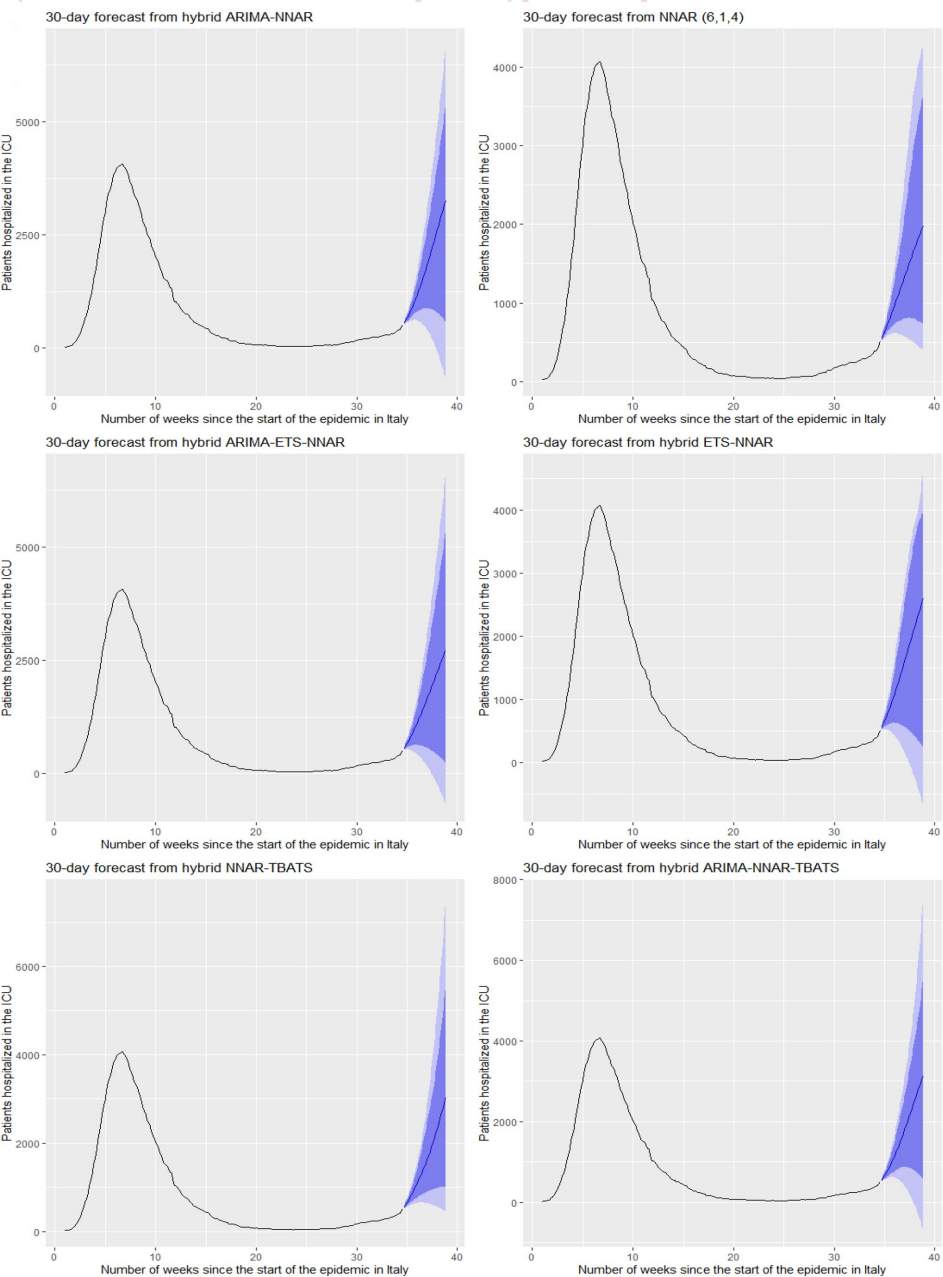
The six best forecast models for predicting patients hospitalized in the ICU. Notes: the models were ranked (from first to sixth place) on the MAE metric

**Fig. 8:**
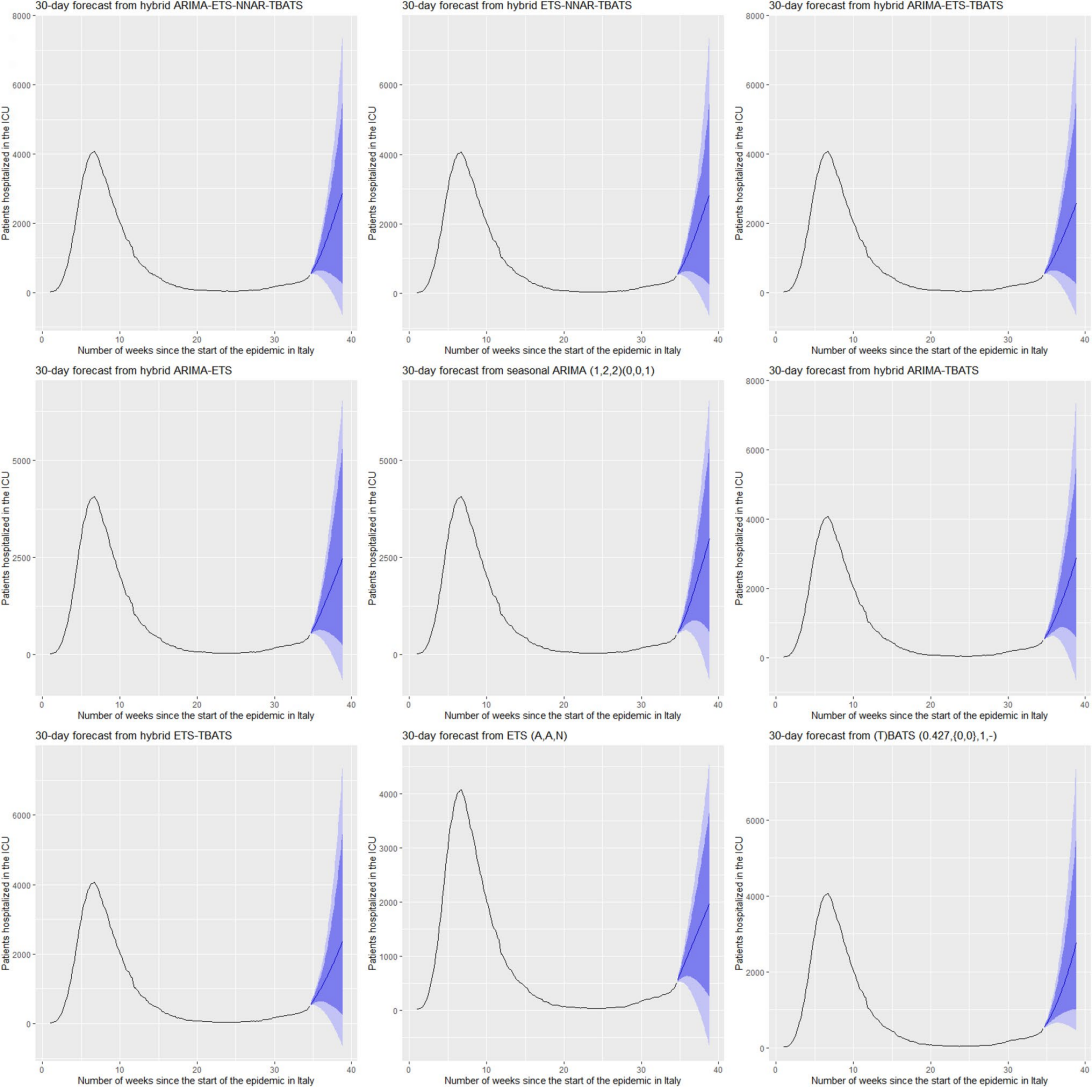
The remaining nine forecast models for predicting patients hospitalized in the ICU. Notes: the models were ranked (from seventh to fifteenth place) on the MAE metric

Figures 5 and 6 show the best six models and the remaining nine models for patients hospitalized with mild symptoms, respectively. Similarly, Figs. 7 and 8 show the best six models and the remaining nine models for patients hospitalized in the ICU, respectively. The light blue area in each graph shows the prediction intervals at 80%, while the dark blue area shows the prediction intervals at 95%.^24^ The forecasts of the best single and hybrid models anticipated an increase in the number of patients hospitalized with mild symptoms and in the number of patients admitted to the ICU over the next 30 days, i.e., from October 14, 2020, to November 12, 2020. This predicted trend was also confirmed by the remaining estimated models.

Specifically, the NNAR–TBATS, ARIMA–NNAR, and ARIMA–NNAR–TBATS models predicted that: (i) after 10 days (October 23), the number of patients hospitalized with mild symptoms should have been 9624, 9397, and 9259, respectively; (ii) after 20 days (by November 2), the number should have been 18,000, 16,986, and 16,062, respectively; and (iii) after 30 days (by November 12), the number should have been 25,039, 21,669, and 21,430, respectively (Fig. 5). Regarding the number of patients hospitalized in the ICU, the ARIMA–NNAR, NNAR, and ARIMA–ETS–NNAR models predicted that: (i) after 10 days, the required number of intensive care beds should have been 1175, 972, and 1114, respectively; (ii) after 20 days, the required number should have been 2164, 1493, and 1915, respectively; and (iii) after 30 days, the number should have been 3270, 1985, and 2726, respectively (Fig. 7).^25^

**Fig. 9:**
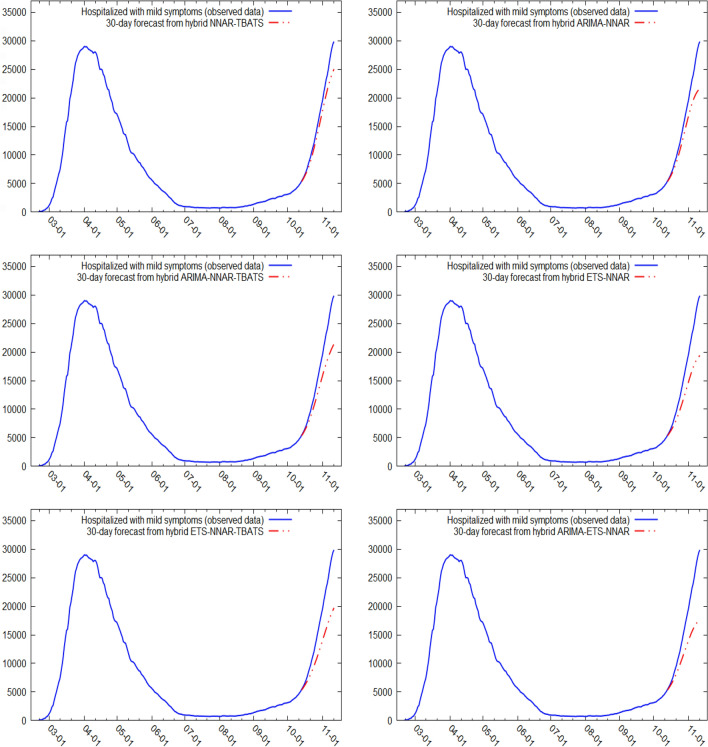
Comparison between forecasts and real data during the period October 14, 2020, to November 12, 2020, for patients hospitalized with mild symptoms (six best models)

**Fig. 10:**
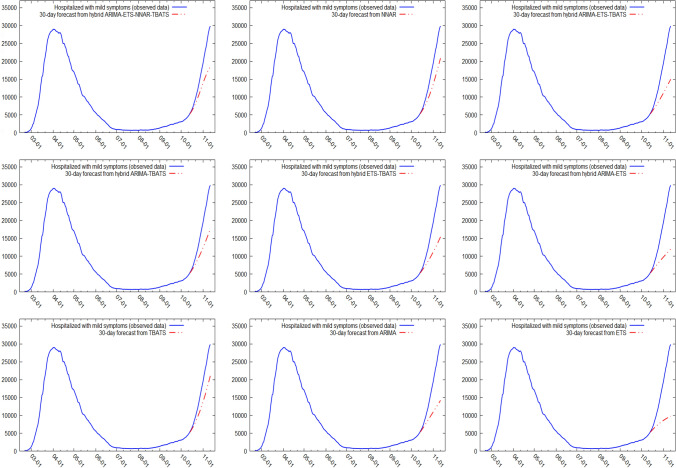
Comparison between forecasts and real data during the period October 14, 2020, to November 12, 2020, for patients hospitalized with mild symptoms (nine remaining models)

**Fig. 11:**
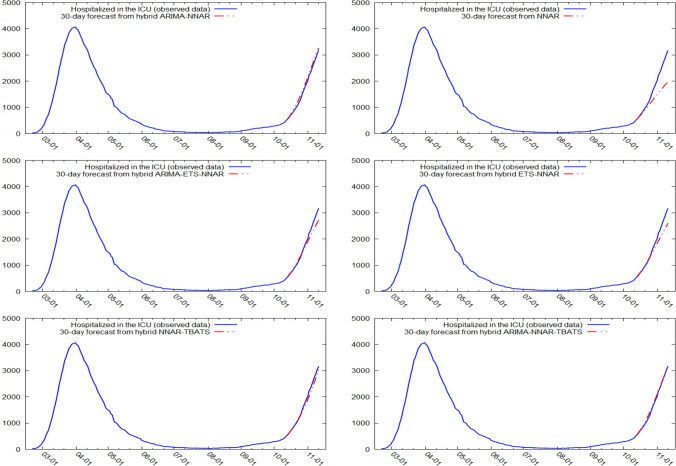
Comparison between forecasts and real data during the period October 14, 2020, to November 12, 2020, for patients hospitalized in the ICU (six best models)

**Fig. 12:**
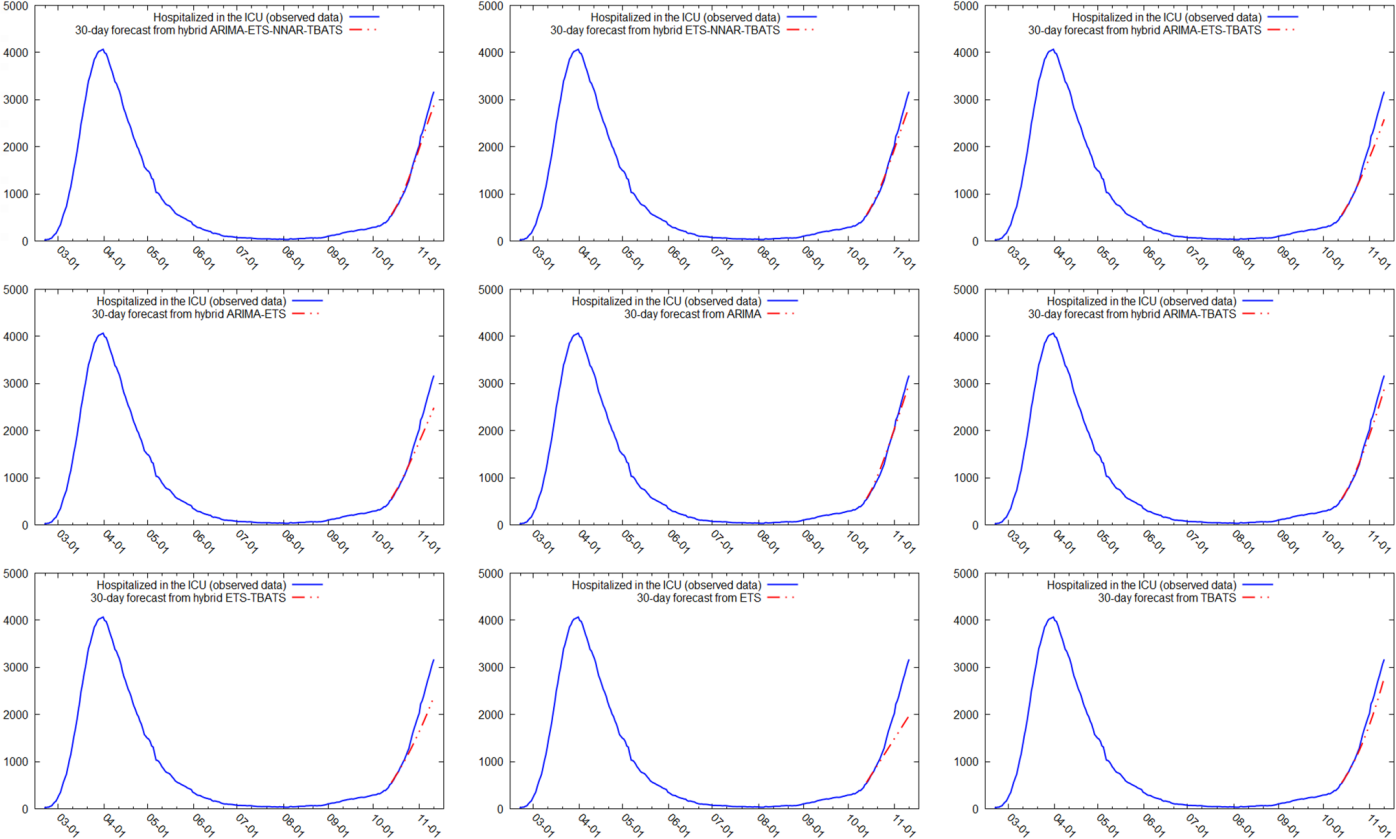
Comparison between forecasts and real data during the period October 14, 2020, to November 12, 2020, for patients hospitalized in the ICU (nine remaining models)

Figures 9, 10, 11 and 12 compare all 15 of the estimated models and the observed data over the period October 14, 2020, to November 12, 2020. For patients hospitalized with mild symptoms, the NNAR–TBATS models best fit the observed data (Fig. 9). Of the remaining models, predictions from ARIMA–NNAR, ARIMA–NNAR–TBATS, and NNAR most closely approximated the observed data (Figs. 9, 10). For patients hospitalized in the ICU, Fig. 11 reveals that ARIMA–NNAR, NNAR–TBATS, and ARIMA–NNAR–TBATS hybrid models also fit the observed data quite well. All estimated models generally exhibited a strong match between predictions and observed data, except for ETS, NNAR, and ETS–TBATS, where the trends differed (Figs. 11 and 12). Notably, the models with lowest loss of efficiency adapted better to the observed data, which confirms the consistency and robustness of the statistical approach.

Thus, a second wave of COVID-19 was predicted for the period following October 13, 2020, which could have several policy implications both for the national healthcare system and the economy. In particular, the predictions underscored the importance of implementing adequate containment measures and increasing the number of ordinary and intensive care beds, hiring additional healthcare personnel, and buying care facilities, protective equipment, and ventilators to fight the infection and reduce deaths.^26^ Meanwhile, the opportunity to implement more or less restrictive non-pharmaceutical interventions (NIPs) to tackle the pandemic—such as social distancing, travel bans, the use of face masks, hand hygiene, and bar and restaurant restrictions [20]—should be evaluated carefully in light of these measures’ potentially negative economic impacts. In fact, according to Fitch Rating’s [24] previsions, the first wave of COVID-19 and the consequent massive lockdown measures may already have caused up to a 9.5% contraction in Italy’s 2020 GDP.

While the models with the lowest loss of efficiency seemed to adapt substantially well to the observed data in the forecast window, the predictions should, in general, be treated with caution and employed mainly to inform short-term decisions. In fact, pandemic forecasting has raised many doubts in the last year due to several issues that can affect its accuracy and reliability, including, for example, (i) high interval predictions and sensitivity of the estimates, especially with long-term forecasts; (ii) inaccurate modeling assumptions, and (iii) the lack of or difficulty in measuring and identifying biological features of COVID-19 transmission [30, 42]. These limitations—and the possibility for misleading forecasts—may erode public trust in science and thus affect compliance with policies intended to mitigate the spread of COVID-19 [49]. Indeed, the inevitable uncertainty associated with this novel disease and the general failure of long-term and even mid-term forecasts require a different scientific approach toward model predictions. More prudent and balanced communication with the public is crucial if the field of science desires to maintain its leading role in human development and policymaking.

## Conclusions

This paper attempted to forecast the short-term dynamics of real-time patients hospitalized from COVID-19 in Italy. In particular, it employed both single time series forecast methods and their feasible hybrid combinations. The results demonstrated that (i) the best single models were NNAR and ARIMA for both patients hospitalized with mild symptom and patients admitted to the ICU, (ii) the most accurate hybrid models were NNAR–TBATS, ARIMA–NNAR, and ARIMA–NNAR–TBATS for patients hospitalized with mild symptoms and ARIMA–NNAR, ARIMA–ETS–NNAR, and ETS–NNAR for patients hospitalized in the ICU, (iii) hybrid models generally outperformed the respective single models by offering more accurate predictions, and (iv) finally, predictions for the number of patients hospitalized in the ICU generally better fit the observed data than did predictions for patients hospitalized with mild symptoms. Notably, the best hybrid models always included a NNAR process, confirming the extensive and successful use of this algorithm in the COVID-19 related literature [56, 57, 71, 72, 81].

Compared to the single models, the hybrid statistical models captured a greater number of properties in the data structure, and the predictions seemed to offer useful policy implications. In fact, consistent with real-time data, the models predicted that the number of patients hospitalized with mild symptoms and admitted to the ICU would grow significantly until mid-November 2020. According to the estimations, the necessary ordinary and intensive care beds were expected to double in 10 days and to triple in approximately 20 days. Thus, since new waves of COVID-19 infections cannot be excluded, it may be necessary to strengthen the national healthcare system by buying protective equipment and hospital beds, managing healthcare facilities, and training healthcare staff.

Although the hybrid models proved to be sufficiently accurate, it is nevertheless important to stress that statistical methods may lead to unavoidable uncertainty and bias, which tend to grow over time, due, for example, to public authorities’ progressive implementation of NIPs, such as the closure of public spaces and national or local lockdown measures, which the forecasts cannot adequately incorporate. Combining hybrid models with mechanistic mathematical models may partially overcome these issues by considering the effects of lockdowns on epidemiological parameters [68]. Thus, future research could proceed along these lines. Ultimately, since other factors may have affected COVID-19 dynamics, especially as the pandemic progressed, these predictions should be treated with caution and utilized only to inform short-term decision-making processes.

## Data Availability

I extracted the data from the official Italian Ministry of Health’s website (www.salute.gov.it).

## Appendix A

See Tables A1, A2 and A3.

**Table A1:** The parameter values of the ARIMA models for patients hospitalized with mild symptoms and in the ICU

Parameters	Coefficients (mild symptoms)	Coefficients (ICU)
AR (1)	0.5963*** [0.1675]	0.8857*** [0.0542]
MA (1)	− 1.0742*** [0.1737]	− 1.4961*** [0.0829]
MA (2)	− 0.0392 [0.1492]	0.6093*** [0.07]
MA (3)	0.3376*** [0.0685]	
Seasonal MA (1)	0.2226*** [0.0742]	0.2707*** [0.0725]

Notes: standard errors in brackets. ****p* value < 0.01

**Table A2:** The parameters values of the ETS models for patients hospitalized with mild symptoms and in the ICU

Parameters	Coefficients (mild symptoms)	Coefficients (ICU)
Smoothing parameters		
*α*	0.9999	0.9071
*Β*	0.4553	0.5871
*ϕ*	0.9709	
Initial states		
*l*	− 182.0547	20.9541
*b*	117.7546	3.4623

**Table A3:** The parameters values of the TBATS models for patients hospitalized with mild symptoms and in the ICU

Parameters	Coefficients (mild symptoms)	Coefficients (ICU)
λ	0.428	0.4267
α	0.2345	0.8384
Β	0.0794	0.371
Damping parameter	1	1
${\gamma }_{1}$	− 0.000198	
${\gamma }_{2}$	− 0.000184	
AR (1)	0.1077	
AR (2)	0.6249	
MA (1)	0.8727	
MA (2)	0.0074	

## Appendix B

See Tables B1, B2, B3 and B4.

**Table B1:** Forecast accuracy measures of the single and hybrid models for patients hospitalized with mild symptoms

Models	MAE	MAPE	RMSE	ACF1
ARIMA–ETS	115.344	3.4616	207.6742	0.0266
ARIMA–NNAR	109.0832	2.2002	191.1117	− 0.0571
ARIMA–TBATS	113.3802	2.6759	200.8599	0.0963
ETS–NNAR	113.8125	2.159	204.3447	0.0091
ETS–TBATS	114.5352	3.396	203.9591	0.0938
NNAR–TBATS	110.5257	2.0911	193.8955	0.1055
ARIMA–ETS–NNAR	111.9864	2.1656	200.0585	− 0.0109
ARIMA–ETS–TBATS	113.0631	3.1329	202.0993	0.0639
ARIMA–NNAR–TBATS	110.9721	2.1299	196.0419	0.0602
ETS–NNAR–TBATS	112.0909	2.0855	199.0529	0.0562
ARIMA–ETS–NNAR–TBATS	111.9338	2.1212	199.1672	0.0369

Notes: Models were combined using cross-validation errors. MASE is omitted because “cv.errors” function currently does not support it.

**Table B2:** Forecast accuracy measures of the single and hybrid models for patients hospitalized in the ICU

Models	MAE	MAPE	RMSE	ACF1
ARIMA–ETS	12.63	3.4796	21.7734	0.0064
ARIMA–NNAR	11.7107	3.0801	19.3024	− 0.0314
ARIMA–TBATS	13.0852	3.4932	22.3627	0.1946
ETS–NNAR	12.4014	3.112	20.7011	− 0.0554
ETS–TBATS	13.3746	3.5436	22.9559	0.1498
NNAR–TBATS	12.8183	2.9997	21.5416	0.1691
ARIMA–ETS–NNAR	12.2468	3.0877	20.7276	− 0.0316
ARIMA–ETS–TBATS	12.9046	3.4813	22.2089	0.1127
ARIMA–NNAR–TBATS	12.465	3.0191	21.0507	0.1209
ETS–NNAR–TBATS	12.7829	3.0591	21.7172	0.0892
ARIMA–ETS–NNAR–TBATS	12.62	3.0555	21.4791	0.0732

Notes: Models were combined using cross-validation errors. MASE is omitted because “cv.errors” function currently does not support it

**Table B3:** Comparison between hybrid models derived using equal weights and weighted errors considering minimization of MAE, MAPE and RMSE (patients hospitalized with mild symptoms)

Models	MAE (%)	MAPE (%)	RMSE (%)
ARIMA–ETS	− 0.22	− 1.68	− 0.27
ARIMA–NNAR	− 3.37	− 1.67	− 3.53
ARIMA–TBATS	*0.14*	− 0.5	− 0.02
ETS–NNAR	− 5.65	− 1.72	− 6.51
ETS–TBATS	*0.16*	*1.95*	*0.31*
NNAR–TBATS	− 5.21	*0.26*	− 5.14
ARIMA–ETS–NNAR	− 2.97	− 1.09	− 3.19
ARIMA–ETS–TBATS	− 0.04	*0.14*	*0.11*
ARIMA–NNAR–TBATS	− 3.89	− 0.21	− 3.59
ETS–NNAR–TBATS	− 3.36	− 0.24	− 3.14
ARIMA–ETS–NNAR–TBATS	− 2.78	− 0.5	− 2.51

Notes: roman values indicated that equal weights were better, while italic values indicate that weighted errors were better.

**Table B4:** Comparison between hybrid models derived using equal weights and weighted errors considering minimization of MAE, MAPE and RMSE (patients hospitalized in the ICU)

Model	MAE (%)	MAPE (%)	RMSE (%)
ARIMA–ETS	− 0.72	− 0.28	− 1.04
ARIMA–NNAR	− 1.87	− 1.16	− 2.87
ARIMA–TBATS	− 1.74	− 0.17	− 1.83
ETS–NNAR	− 4.01	− 1.54	− 5.56
ETS–TBATS	− 0.01	−	*0.01*
NNAR–TBATS	− 6.5	− 0.77	− 8.57
ARIMA–ETS–NNAR	− 2.95	− 0.91	− 4.25
ARIMA–ETS–TBATS	− 3.23	− 3.47	− 3.65
ARIMA–NNAR–TBATS	− 3.69	− 0.33	− 5.07
ETS–NNAR–TBATS	− 3.13	− 0.75	− 4.39
ARIMA–ETS–NNAR–TBATS	− 3.42	− 0.98	− 4.38

Notes: roman values indicated that equal weights were better, while italic colored values indicated that weighted errors were better.

## Appendix C

See Tables C1 and C2.

**Table C1:** The Ljung–Box’s test results for autocorrelation in the models (patients hospitalized with mild symptoms)

Models MILD Symptoms	Lags 2	Lags 5	Lags 8	Lags 10
ARIMA	0.8093	0.437	0.6336	0.6986
ETS	0.1105	0.0157	0.001	0.0022
NNAR	0.0993	0.1274	0.0003	0.001
TBATS	0.6925	0.9045	0.5567	0.7197
A–E	0.1102	0.1444	0.2007	0.281
A–N	0.2038	0.2678	0.1012	0.1296
A–T	0.2129	0.3416	0.5749	0.6488
E–N	0.0519	0.0991	0.0139	0.044
E–T	0.0091	0.0398	0.0737	0.07
N–T	0.2428	0.2627	0.1088	0.1514
A–N–T	0.4573	0.4601	0.3396	0.4048
A–E–N	0.074	0.254	0.1631	0.229
A–E–T	0.0938	0.1763	0.2701	0.3763
E–N–T	0.0518	0.0986	0.0535	0.0925
A–E–N–T	0.1105	0.2543	0.2225	0.3068

Notes: *A* ARIMA, *E* ETS, *N* NNAR, *T* TBATS

**Table C2:** The Ljung–Box’s test results for autocorrelation in the models (patients hospitalized in the ICU)

Models ICU	Lags 2	Lags 5	Lags 8	Lags 10
ARIMA	0.2477	0.3673	0.5745	0.7385
ETS	0.1565	0.063	0.0502	0.000
NNAR	0.0713	0.1089	0.0004	0.0014
TBATS	0.3775	0.5861	0.002	0.0052
A–E	0.1415	0.2366	0.0652	0.1353
A–N	0.0632	0.1105	0.11	0.2279
A–T	0.0985	0.0618	0.000	0.0000
E–N	0.0648	0.1036	0.1591	0.001
E–T	0.0199	0.0006	0.0000	0.0000
N–T	0.2137	0.7283	0.8108	0.0041
A–N–T	0.1884	0.6286	0.0682	0.1397
A–E–N	0.0959	0.2094	0.0787	0.174
A–E–T	0.1257	0.0755	0.0002	0.0004
E–N–T	0.1832	0.5328	0.6055	0.0505
A–E–N–T	0.1807	0.5352	0.0549	0.0673

Notes: *A* ARIMA, *E* ETS, *N* NNAR, *T* TBATS

## Appendix D

See Tables D1 and D2.

**Table D1:** The predicted values of patients hospitalized with mild symptoms in Italy, from October 14, 2020 to November 12, 2020 (six best models)

Date	Patients hospitalized with mild symptoms					
	N–T	A–N	A–N–T	E–N	E–N–T	A–E–N
14-10-2020	5336.86	5347.71	5337.33	5338.38	5324.25	5336.2
15-10-2020	5651.95	5654.09	5644.54	5619.01	5602.82	5619.39
16-10-2020	6010.62	5990.28	5987.29	5916.08	5906	5921.66
17-10-2020	6393.93	6361.31	6358.13	6229.25	6218.18	6246.44
18-10-2020	6812.83	6766.48	6750.66	6573.92	6.548.27	6593.6
19-10-2020	7276.66	7209.03	7183.44	6934.69	6900.21	6965.03
20-10-2020	7779.26	7687.39	7643.35	7324.16	7268.47	7359.22
21-10-2020	8321.9	8209.49	8131.61	7746.5	7653.02	7781.47
22-10-2020	8932.9	8779.31	8668.44	8202.94	8071.92	8233.28
23-10-2020	9624.15	9397.23	9259.27	8698.68	8531.95	8713.3
24-10-2020	10,357.4	10,061.49	9877.39	9235.54	9012.85	9218.5
25-10-2020	11,117.49	10,764.26	10,511.98	9805.32	9510.84	9740.9
26-10-2020	11,917.57	11,500.99	11,168.61	10,401.09	10,039.3	10,275.97
27-10-2020	12,733.97	12,264.65	11,834.96	11,013.84	10,585.59	10,819.94
28-10-2020	13,550.94	13,047.27	12,508.36	11,637.78	11,139.26	11,370.28
29-10-2020	14,406.58	13,842.35	13,212.81	12,272.34	11,723.14	11,925.53
30-10-2020	15,305.39	14,642.62	13,942.71	12,919.64	12,338.97	12,482.49
31-10-2020	16,202.31	15,439.95	14,660.76	13,581.66	12,955.84	13,037.2
1-11-2020	17,093.01	16,224.71	15,361.98	14,252.71	13,571	13,585.9
2-11-2020	17,999.69	16,985.92	16,061.7	14,919.48	14,200.33	14,124.03
3-11-2020	18,888.69	17,712.48	16,739.24	15,570.64	14,826.46	14,645.62
4-11-2020	19,736.72	18,393.74	17,376.33	16,198.52	15,435.74	15,143.07
5-11-2020	20,573.69	19,020.32	17,996.11	16,794.27	16,051.78	15,608.2
6-11-2020	21,397.74	19,585.29	18,602.42	17,346.33	16,674.35	16,033.46
7-11-2020	22,153.42	20,085.04	19,162.8	17,843.65	17,265.48	16,413.91
8-11-2020	22,837.29	20,519.42	19,677.54	18,279.44	17,820.17	16,749.2
9-11-2020	23,480.72	20,890.96	20,169.1	18,651.09	18,355.65	17,041.07
10-11-2020	24,059.64	21,203,59	20,622.78	18,958.25	18,852	17,291.64
11-11-2020	24,560.39	21,461.63	21,030.51	19,202.09	19,296.82	17,503.69
12-11-2020	25,038.65	21,669.2	21,429.73	19,385.22	19,724.79	17,680.5

Notes: *A*  ARIMA, *E*  ETS, *N*  NNAR, *T*   TBATS

**Table D2:** The predicted values of patients hospitalized in the ICU in Italy, from October 14, 2020 to November 12, 2020 (six best models)

Date	Patients hospitalized in ICU					
	A–N	N	A–E–N	E–N	N–T	A–N–T
14-10-2020	561.38	552.34	560.63	560.17	556.32	558.3
15-10-2020	609.88	587.43	609.54	607.84	601.59	606.11
16-10-2020	666.55	632.77	663.35	659.77	651.81	660.22
17-10-2020	724.24	678.52	717.5	714.29	705.48	715.95
18-10-2020	788.99	724.27	776.18	770.55	761.81	777.22
19-10-2020	858.45	774.31	838.26	830.93	822.91	842.98
20-10-2020	935.13	822.37	905.24	892.93	885.85	915.27
21-10-2020	1011.28	870.49	971.93	956.76	950.42	988.64
22-10-2020	1091.55	921.35	1041.7	1023.48	1017.24	1066.22
23-10-2020	1174.99	972.36	1113.99	1092.27	1085.73	1147.44
24-10-2020	1261.29	1023.36	1188.17	1162.67	1157.32	1231.88
25-10-2020	1350.75	1075.3	1264.1	1234.94	1231.24	1319.65
26-10-2020	1442.95	1127.25	1341.38	1308.44	1306.6	1410.39
27-10-2020	1537.98	1179.26	1420	1382.99	1383.99	1503.89
28-10-2020	1636.04	1231.7	1500.11	1458.61	1463.84	1600.11
29-10-2020	1736.85	1284.11	1581.46	1534.79	1546.25	1698.67
30-10-2020	1840.25	1336.37	1663.8	1611.31	1631.76	1799.19
31-10-2020	1946.12	1388.63	1747.01	1688.29	1720.53	1901.4
1-11-2020	2054.19	1440.7	1830.84	1765.57	1812.69	2004.94
2-11-2020	2164.21	1492.51	1915.1	1843.02	1908.64	2109.52
3-11-2020	2275.92	1544.08	1999.58	1920.59	2008.61	2214.94
4-11-2020	2388.88	1595.3	2083.98	1998.12	2112.68	2320.88
5-11-2020	2502.67	1646.09	2168.03	2075.54	2220.81	2427.05
6-11-2020	2616.77	1696.44	2251.46	2152.8	2332.7	2533.12
7-11-2020	2730.57	1746.27	2334.01	2229.78	2447.86	2638.73
8-11-2020	2843.41	1795.51	2415.46	2306.34	2565.64	2743.53
9-11-2020	2954.55	1844.11	2495.56	2382.29	2685.18	2847.15
10-11-2020	3063.22	1891.99	2574.1	2457.4	2805.52	2949.16
11-11-2020	3168.58	1939.08	2650.86	2531.37	2925.64	3049.14
12-11-2020	3269.82	1985.3	2725.65	2603.83	3044.52	3146.65

Notes: *A*  ARIMA, *E*  ETS, *N*  NNAR, *T* TBATS

## Footnotes

1 In fact, the number of infected people presents a high degree of uncertainty, especially because the number of infected but asymptomatic people is high [59].

2 For more details see Equations (2) and (3).

3 It is valid only for the trend component.

4 See also, for example, Fallah et al. [21].

5 Both the augmented Dickey–Fuller test [19] and the Kwiatkowsky, Phillips, Schmidt, and Shin test [50] were used. In fact, as stated by Gujarati and Porter [28], there is no recognized uniformly powerful test for detecting unit roots.

6 The AICc is a bias-corrected version of the original Akaike information criterion (AIC) proposed by Sugiura [70] and Hurvich and Tsai [32]. The former performed significantly better than the latter in both small and moderate sample sizes [32]. Thus, it is suitable for this study.

7 A description of the “ets()” function is provided by Hyndman and Athanasopoulos [34], Sect. 7.6).

8 A description of the “nnetar()” function is provided by Hyndman and Athanasopoulos [34], Sect. 11.3).

9 This was the default value for the seasonal time series in the “nnetar()” function.

10 In fact, AICc is not included in the “tbats()” function.

11 A detailed description of the “forecastHybrid” package was provided at https://cran.r-project.org/web/packages/forecastHybrid/forecastHybrid.pdf.

12 To this regard, it is important to stress that Livera et al. [17] introduced a specific trigonometric representation of seasonal components based on Fourier series.

13 Hyndman [37] and Hyndman and Athanasopoulos [34], Sect. 3.4) offer a more detailed explanation of these metrics.

14 The parameter values of the ARIMA, ETS, and TBATS models were reported in Tables A1, A2, and A3 (Appendix A).

15 As suggested by Hyndman [38], since the time series covered less than year and had daily observations, the frequency was set to 7, which allowed for weekly seasonality.

16 The selected ETS model is also known as the damped trend method with additive errors.

17 The estimated TBATS model was derived by applying a Box–Cox transformation of 0.428, an ARMA{2,2} process for modeling errors, a damping parameter of 1 (doing nothing), and two Fourier pairs with seasonal periods of 7.

18 The selected ETS model is also known as Holt’s linear trend method. In this case, both error and trend were additive.

19 In this case, the “tbats()” algorithm gave a BATS model that differed from TBATS only in the way that it modeled seasonality. In particular, it did not implement the Fourier series to model seasonality. For simplicity, the notation TBATS was used for the discussion of hybrid models forecasting patients hospitalized in the ICU. This model employed a Box–Cox transformation of 0.427, no ARMA {0,0} errors, and a damping parameter of 1 (doing nothing).

20 The forecast performance measures of the hybrid models combined with the cross-validation procedure were reported in the Appendix B (Tables B1 and B2). With regard to patients hospitalized with mild symptoms and patients hospitalized in the ICU, the models derived using equal weights outperformed those obtained with cross-validated errors in 26 and 31 (out of 33) performance accuracy metrics, respectively Tables B3 and B4.

21 In particular, ARIMA–ETS–NNAR and ETS–NNAR models exhibited nearly identical accuracy.

22 Tables C1 and C2 (Appendix C) also reported the results of Ljung–Box’s [54] test for residual autocorrelation using no more than 10 lags, as suggested by Ljung [53] when the sample size is not large. The output showed that for patients hospitalized with mild symptoms, ARIMA, TBATS, and all hybrid models, except for ETS-TBATS, had no autocorrelation issue, while single ETS and NNAR exhibited some autocorrelation, especially at lags 8 and 10. With regard to patients hospitalized in the ICU, ARIMA and most of hybrid models, except for ARIMA–TBATS, ETS–TBATS, and ARIMA–ETS–TBATS, had no particular signs of autocorrelation. In contrast, single ETS, NNAR, and TBATS showed some problems at lags 8 and 10.

23 This did not change the meaning of the results because, as confirmed by Table C2 (Appendix C), the residuals of the hybrid model’s output were generally less affected by autocorrelation than were those for NNAR.

24 The choice of these percentages was consistent with Hyndman and Athanasopoulos (34, Sect. 3.5).

25 Values are rounded to the nearest integer. The predicted values of the six best models for patients hospitalized with mild symptoms and in the ICU were reported in Table D1 and D2 (Appendix D), respectively.

26 This is consistent with my recent paper [64] in which I demonstrated that the Italian healthcare system’s saturation played a key role in explaining the variability of COVID-19 mortality. In particular, the saturation of ordinary and intensive care beds explained almost 90% of the COVID-19 mortality across Italian regions at the first peak of the pandemic.

## Code availability

The statistic software and codes are mentioned in the manuscript (“Materials and methods”).
